# Associations of the vasoactive peptides CT-proET-1 and MR-proADM with incident type 2 diabetes: results from the BiomarCaRE Consortium

**DOI:** 10.1186/s12933-022-01513-9

**Published:** 2022-06-09

**Authors:** Chaterina Sujana, Veikko Salomaa, Frank Kee, Jochen Seissler, Pekka Jousilahti, Charlotte Neville, Cornelia Then, Wolfgang Koenig, Kari Kuulasmaa, Jaakko Reinikainen, Stefan Blankenberg, Tanja Zeller, Christian Herder, Ulrich Mansmann, Annette Peters, Barbara Thorand, for the BiomarCaRE Consortium

**Affiliations:** 1 Institute of Epidemiology, Helmholtz Zentrum München - German Research Center for Environmental Health, Ingolstädter Landstrasse 1, 85764 Neuherberg, Germany; 2 Institute for Medical Information Processing, Biometry, and Epidemiology (IBE), Pettenkofer School of Public Health, Ludwig-Maximilians-Universität München, Munich, Germany; 3 German Center for Diabetes Research (DZD), Partner München-Neuherberg, Neuherberg, Germany; 4 Department of Public Health and Welfare, Finnish Institute for Health and Welfare, Helsinki, Finland; 5 Centre for Public Health, Queens University of Belfast, Belfast, Northern Ireland UK; 6 Diabetes Zentrum, Medizinische Klinik Und Poliklinik IV, Klinikum Der Ludwig-Maximilians-Universität München, Munich, Germany; 7 Institute of Epidemiology and Medical Biometry, University of Ulm, Ulm, Germany; 8 Deutsches Herzzentrum München, Technische Universität München, Munich, Germany; 9 German Centre for Cardiovascular Research (DZHK E.V.), Partner Site Munich Heart Alliance, Munich, Germany; 10 Department for General and Interventional Cardiology, University Heart Center Hamburg, Hamburg, Germany; 11 German Centre for Cardiovascular Research (DZHK E.V.), Partner site Hamburg, Lübeck, Kiel, Hamburg, Germany; 12 Institute for Clinical Diabetology, German Diabetes Center, Leibniz Center for Diabetes Research at Heinrich-Heine-University Düsseldorf, Düsseldorf, Germany; 13 Division of Endocrinology and Diabetology, Medical Faculty and University Hospital Düsseldorf, Heinrich-Heine-University Düsseldorf, Düsseldorf, Germany; 14 German Center for Diabetes Research (DZD), Partner Düsseldorf, Neuherberg, Germany

**Keywords:** Adrenomedullin, C-terminal-proendothelin-1, Endothelin-1, Epidemiology, Incident type 2 diabetes, Mendelian randomisation, Mid-regional-proadrenomedullin, Cohort study

## Abstract

**Background:**

Endothelin-1 (ET-1) and adrenomedullin (ADM) are commonly known as vasoactive peptides that regulate vascular homeostasis. Less recognised is the fact that both peptides could affect glucose metabolism. Here, we investigated whether ET-1 and ADM, measured as C-terminal-proET-1 (CT-proET-1) and mid-regional-proADM (MR-proADM), respectively, were associated with incident type 2 diabetes.

**Methods:**

Based on the population-based Biomarkers for Cardiovascular Risk Assessment in Europe (BiomarCaRE) Consortium data, we performed a prospective cohort study to examine associations of CT-proET-1 and MR-proADM with incident type 2 diabetes in 12,006 participants. During a median follow-up time of 13.8 years, 862 participants developed type 2 diabetes. The associations were examined in Cox proportional hazard models. Additionally, we performed two-sample Mendelian randomisation analyses using published data.

**Results:**

CT-proET-1 and MR-proADM were positively associated with incident type 2 diabetes. The multivariable hazard ratios (HRs) [95% confidence intervals (CI)] were 1.10 [1.03; 1.18], P = 0.008 per 1-SD increase of CT-proET-1 and 1.11 [1.02; 1.21], P = 0.016 per 1-SD increase of log MR-proADM, respectively. We observed a stronger association of MR-proADM with incident type 2 diabetes in obese than in non-obese individuals (P-interaction with BMI < 0.001). The HRs [95%CIs] were 1.19 [1.05; 1.34], P = 0.005 and 1.02 [0.90; 1.15], P = 0.741 in obese and non-obese individuals, respectively. Our Mendelian randomisation analyses yielded a significant association of CT-proET-1, but not of MR-proADM with type 2 diabetes risk.

**Conclusions:**

Higher concentrations of CT-proET-1 and MR-proADM are associated with incident type 2 diabetes, but our Mendelian randomisation analysis suggests a probable causal link for CT-proET-1 only. The association of MR-proADM seems to be modified by body composition.

**Supplementary Information:**

The online version contains supplementary material available at 10.1186/s12933-022-01513-9.

## Background

Metabolic and vascular diseases commonly coexist. However, the pathophysiological mechanisms linking both diseases are not well understood. A possible link is the dysregulation of vasoactive peptides that could be implicated in both vascular and metabolic homeostasis [1], such as endothelin-1 (ET-1) and adrenomedullin (ADM).

ET-1, a 21-amino acid peptide primarily secreted by vascular endothelial cells, is a potent vasoconstrictor and pro-inflammatory peptide [2]. ET-1 has been implicated in the pathogenesis of several chronic diseases, including hypertension and chronic kidney disease [3]. In addition to its known effect on the vascular function, ET-1 also limits insulin actions in skeletal muscles and adipocytes leading to insulin resistance and impaired glucose tolerance [4–6]. ADM, a 52-amino acid peptide that belongs to the calcitonin gene–related peptide family, is a vasodilator. ADM is secreted by a variety of different cells, including vascular endothelial cells, smooth muscle cells, adventitial fibroblasts as well as adipocytes [7, 8]. In a later investigation, adipose tissue was suggested to be the major source of ADM [8]. ADM has several metabolic actions, including counteracting oxidative stress–induced insulin resistance [9, 10] and inhibition of insulin secretion from the pancreatic islets [11].

Measurements of circulating concentrations of ET-1 and ADM are very difficult because of the short half-life, the existence of binding proteins, and other technical difficulties. Therefore, assays have been developed to measure the inactive fragments of ET-1 and ADM as the surrogates, C-terminal-proendothelin-1 (CT-proET-1) and mid-regional-proadrenomedullin (MR-proADM), respectively, which are biologically stable and are correlated with the active peptides in equimolar concentrations [12, 13].

In epidemiological studies using a cross-sectional design, both CT-proET-1 and MR-proADM were positively associated with the metabolic syndrome, insulin resistance and prevalent type 2 diabetes [14–16]. Previously, using a prospective study design, we have also shown that higher circulating concentrations of CT-proET-1 and MR-proADM were associated with increased insulin resistance, suggesting that both vasoactive peptides could play a role in the pathogenesis of type 2 diabetes [17]. However, most of the existing prospective studies failed to provide evidence for an association of both CT-proET-1 and MR-proADM with incident type 2 diabetes [17–20]. So far, only two prospective studies reported a positive association between CT-proET-1 and incident type 2 diabetes [21, 22]. Thus, we aimed to examine the putative association of CT-proET-1 and MR-proADM with incident type 2 diabetes by performing a prospective cohort study with a larger sample size using data from the multinational Biomarkers for Cardiovascular Risk Assessment in Europe (BiomarCaRE) Consortium [23] in tandem with a two-sample Mendelian randomisation study using published data on genetic variants that are specific for CT-proET-1 or MR-proADM, to allow a more robust analysis.

## Methods

### Study population

BiomarCaRE is an EU-funded consortium that aims to determine the value of established and emerging biomarkers in improving risk estimation of cardiovascular disease. BiomarCaRE relies on the Monitoring of Trends and Determinants in Cardiovascular Diseases (MONICA) Risk Genetics Archiving and Monograph (MORGAM) Project [24], which includes harmonized data from a large number of population-based cohorts. All participating cohorts were approved by local ethical review boards and written informed consent was obtained from all study participants. The study was conducted according to the Declaration of Helsinki.

In the prospective cohort study, we included three BiomarCaRE population-based cohorts involving 12,006 participants initially without diabetes and cardiovascular diseases and with follow-up data on type 2 diabetes. The exclusion criteria are described in Additional file 1: Fig. S1. The participating cohorts were the FINRISK Study (Finland), the Prospective Epidemiological Study of Myocardial Infarction (PRIME) Belfast (UK), and the Cooperative Health Research in the Region of Augsburg Study (KORA) F4 (Germany). An overview of each participating cohort is provided in Additional file 1: Table S1. The following harmonized variables were available for each cohort: age, sex, body mass index (BMI), waist circumference, systolic and diastolic blood pressure, antihypertensive medication, smoking status, total and high-density lipoprotein (HDL) cholesterol and diabetes status.

### Ascertainment of type 2 diabetes cases

We defined prevalent diabetes as a documented diagnosis of diabetes at baseline, either identified by record linkage or through self-report of the participants that were verified by medical chart review or through information obtained from the treating physician. Incident type 2 diabetes was defined as a new diagnosis of type 2 diabetes during follow-up, either identified by record linkage or through self-report of the participants initially without diabetes at baseline that were verified by medical record review or through information obtained from the treating physician. Details of the assessment of type 2 diabetes in each participating cohort are provided in Additional file 1: Table S1.

### Laboratory measurements

Baseline concentrations of CT-proET-1 and MR-proADM were measured from plasma with immunoluminometric assay (BRAHMS/Thermo Fisher Scientific, Hennigsdorf, Berlin, Germany) on the BRAHMS KRYPTOR automated system. The data were measured centrally in the MORGAM/BiomarCaRE core laboratory for FINRISK and PRIME Belfast (in 2008) and locally for KORA F4 (in 2010). The cohort-specific intra- and interassay coefficients of variation for CT-proET-1 and MR-proADM are described in Additional file 1: Table S2. Laboratory procedures for other diabetes-related biomarkers used in the analyses are provided in Additional file 1: Text S1.

### Statistical analysis

Measurement values below the limit of detection (LOD) (N = 121 for CT-proET-1 and N = 166 for MR-proADM, all from the FINRISK study) were set to the lower LOD (i.e. 9.44 pmol/l for CT-proET-1 and 0.24 nmol/l for MR-proADM). Other missing values of the vasoactive peptides or missing values of diabetes risk factors (Additional file 1: Table S3) were handled with multiple imputation by chained equations (MICE), performed using R package mice [25], version 3.13. The imputation was done separately for each cohort. A total of 200 imputed data sets were created. Additional variation due to imputation was taken into account according to the Rubin’s rules for multiple imputation [26].

Descriptive statistics are reported for the participants stratified by incident type 2 diabetes during follow-up and shown as frequency (percentage) for categorical variables and as mean (standard deviation (SD)) for continuous variables. Continuous variables with skewed distributions are presented as geometric mean (antilog SD).

The associations of both CT-proET-1 and MR-proADM with incident type 2 diabetes were estimated by calculating hazard ratios (HRs) with 95% confidence intervals (95% CIs) in Cox proportional hazard (PH) models. The models were stratified by study cohort and were adjusted for age (continuous, in years) and sex (men/women) in model 1 and were further adjusted for current smoking (yes/no), total and HDL cholesterol (continuous, in mmol/l), actual hypertension (yes/no) and BMI (continuous, in kg/m^2^) in model 2. Actual hypertension was defined as having systolic blood pressure ≥ 140 mmHg, diastolic blood pressure ≥ 90 mmHg or using antihypertensive medication. The distribution of MR-proADM was right-skewed (Additional File 1: Fig. S2) and thus was log-transformed to approximate normality. Both peptides were (0,1)-standardized to estimate the HRs per 1-SD increase. To further evaluate whether other diabetes-related biomarkers might account for the observed associations, we additionally included the baseline measurement of estimated glomerular filtration rate (eGFR), insulin, high-sensitivity C-reactive protein (hsCRP), leptin, and fasting glucose individually and simultaneously in model 2. The PH assumption was tested by plotting scaled Schoenfeld residuals against follow-up time for each covariate. No indication of non-proportionality was observed.

We tested for interactions of both peptides with BMI, sex and actual hypertension by creating cross-product terms and evaluating the significance level. Additionally, we also tested for the interaction with waist circumference as an alternative to BMI. False discovery rate (FDR) with the Benjamini–Hochberg method was used to correct for multiple testing. An interaction was considered relevant at FDR < 0.05. Subgroup analyses were conducted by examining the associations across BMI (≥ 30 kg/m^2^ vs < 30 kg/m^2^), waist circumference (men: ≥ 102 cm, women: ≥ 88 cm vs men: < 102, women: < 88 cm), sex (men vs women) and actual hypertension (yes vs no) categories. We also calculated the associations of CT-proET-1 and MR-proADM with incident type 2 diabetes for each participating cohort. Heterogeneity in the association across cohorts were examined by testing the interaction by study cohort and by examining Cochran’s Q and I^2^.

To examine the associations of genetically predicted CT-proET-1 and MR-proADM with type 2 diabetes risk, we performed two-sample univariate Mendelian randomisation analyses using results from published genome-wide association (GWA) studies. We identified single nucleotide polymorphisms (SNPs) with effects specific to either CT-proET-1 or MR-proADM at a *P*-value < 5E-8 as the genetic instrumental variables (IVs) from a published GWA study of European ancestry from Verweij, et al. [27]. Estimates of the genetic association with type 2 diabetes were extracted from meta-analyses of GWA studies for populations of European ancestry by Mahajan et al. [28] and Bonàs-Guarch et al. [29], depending on the data availability. The procedure for the Mendelian randomisation analysis is provided in detail in Additional file 1: Text S2.

All statistical analyses were performed using R version 4.0.3 [30]. *P*-values less than 0.05 were considered statistically significant.

## Results

### Baseline characteristics of study participants

Baseline characteristics of participants according to incident type 2 diabetes status during follow-up are summarized in Table 1. During a median follow-up time of 13.8 years (interquartile range of 4.8), 862 out of 12,006 participants developed type 2 diabetes. Participants who developed type 2 diabetes were more frequently men. At baseline, in comparison to non-cases, the cases of incident type 2 diabetes were on average older, had higher concentrations of CT-proET-1 and MR-proADM, had a higher BMI and waist circumference, were more frequently hypertensive, had lower eGFR, had lower concentrations of HDL cholesterol and higher concentrations of total cholesterol, hsCRP, insulin and leptin. Participant characteristics for each participating cohort are presented in Additional file 1: Table S4.

**Table 1:** Participant characteristics in the total study population and stratified by incident type 2 diabetes status

	Overall	Incident type 2 diabetes	
		Cases	Non-cases
Number of individuals	12,006	862	11,144
Cohort (N (%))			
FINRISK	7336 (61.1)	531 (61.6)	6805 (61.1)
PRIME Belfast	2496 (20.8)	240 (27.8)	2256 (20.2)
KORA F4	2174 (18.1)	91 (10.6)	2083 (18.7)
CT-proET-1, in pmol/l [mean (SD)]	50.7 (13.4)	55.5 (14.2)	50.3 (13.3)
MR-proADM, in nmol/l [geometric mean (antilog SD)]	0.46 (1.31)	0.52 (1.30)	0.45 (1.30)
Age, in years [mean (SD)]	49.4 (11.8)	54.7 (9.2)	49.0 (11.8)
Male [N (%)]	7072 (58.9)	615 (71.3)	6457 (57.9)
Body mass index, in kg/m^2^ [mean (SD)]	26.5 (4.25)	30.6 (5.04)	26.1 (4.01)
Waist circumference, in cm [mean (SD)]	88.9 (12.8)	101 (12.9)	87.9 (12.3)
Actual hypertension [N (%)]^a^	4899 (40.8)	608 (70.5)	4,291 (38.5)
Systolic blood pressure, in mmHg [mean (SD)]	132.1 (20.1)	144.0 (20.7)	131.2 (19.8)
Diastolic blood pressure, in mmHg [mean (SD)]	81.0 (11.3)	87.2 (11.4)	80.5 (11.2)
Use of antihypertensive medication [N (%)]	1,308 (10.9)	225 (26.1)	1,083 (9.7)
Current smoker [N (%)]	3,234 (26.9)	234 (27.1)	3,000 (26.9)
Total cholesterol, in mmol/l [mean (SD)]	5.59 (1.05)	5.89 (1.06)	5.56 (1.05)
HDL, in mmol/l [mean (SD)]	1.37 (0.37)	1.19 (0.33)	1.39 (0.37)
eGFR (ml/min/1.73m^2^) [mean (SD)]	89.1 (19.4)	84.8 (21.3)	89.4 (19.2)
Insulin (microU/ml) [geometric mean (antilog SD)]	5.79 (1.85)	9.04 (1.89)	5.60 (1.82)
hsCRP (mg/l) [geometric mean (antilog SD)]	1.23 (3.03)	2.19 (2.83)	1.18 (3.01)
Leptin (ng/ml) [geometric mean (antilog SD)]	7.30 (2.69)	11.07 (2.52)	7.07 (2.69)
Fasting glucose (mmol/l) [geometric mean (antilog SD)]^b^	5.01 (1.13)	5.45 (1.23)	4.98 (1.12)

Data are presented as frequency (percentage) for categorical variables and as mean (SD) for continuous variables. Continuous variables with skewed distributions are presented as geometric mean (antilog SD)

*CT-proET-1* C-terminal-proendothelin-1, *eGFR* estimated glomerular filtration rate, *HDL* high-density lipoprotein, *hsCRP* high-sensitivity C-reactive protein, *KORA* Cooperative Health Research in the Region of Augsburg Study, *MR-proADM* mid-regional-proadrenomedullin, *PRIME* Prospective Epidemiological Study of Myocardial Infarction, *SD* standard deviation

^a^Actual hypertension was defined as having systolic blood pressure ≥ 140 mmHg, diastolic blood pressure ≥ 90 mmHg or using antihypertensive medication

^b^Data were available and calculated in 9112 participants of FINRISK and KORA F4 who fasted at least 4 h (593 cases and 8519 non-cases of incident type 2 diabetes)

### Associations of CT-proET-1 and MR-proADM with incident type 2 diabetes

Both CT-proET-1 and MR-proADM were positively associated with incident type 2 diabetes in the overall study population. The HRs [95% CIs] in model 1 were 1.30 [1.21; 1.39] per 1-SD increase of CT-proET-1 and 1.57 [1.45; 1.69] per 1-SD increase of log MR-proADM. The associations were attenuated, but remained statistically significant after additional adjustment for diabetes risk factors according to model 2 (HR [95% CI]: 1.10 [1.03; 1.18] per 1-SD increase of CT-proET-1 and 1.11 [1.02; 1.21] per 1-SD increase of log MR-proADM). The association for CT-proET-1 remained stable when we further adjusted for eGFR, insulin, hsCRP, leptin, and fasting glucose (Table 2 and Additional File 1: Table S5). However, it was no longer significant for MR-proADM when insulin, hsCRP or leptin were added to the model (Table 2). The associations of CT-proET-1 and MR-proADM with incident type 2 diabetes were also examined in each participating cohort with a negligible level of heterogeneity (Additional File 1: Figs. S3 and S4, respectively).

**Table 2:** Association of CT-proET-1 and MR-proADM with incident type 2 diabetes

Adjustment	Hazard ratio [95% CI]
	N cases/person-years = 862/149,937
CT-proET-1	
Model 1	1.30 [1.21; 1.39], P < 0.001
Model 2	1.10 [1.03; 1.18], P = 0.008
Model 2 + eGFR	1.10 [1.03; 1.19], P = 0.007
Model 2 + insulin^a^	1.10 [1.02; 1.18], P = 0.012
Model 2 + hsCRP	1.08 [1.01; 1.16], P = 0.026
Model 2 + leptin	1.09 [1.02; 1.17], P = 0.018
Model 2 + eGFR, insulin, hsCRP, leptin	1.09 [1.01; 1.17], P = 0.021
MR-proADM	
Model 1	1.57 [1.45; 1.69], P < 0.001
Model 2	1.11 [1.02; 1.21], P = 0.016
Model 2 + eGFR	1.12 [1.02; 1.22], P = 0.013
Model 2 + insulin^a^	1.09 [1.00; 1.18], P = 0.061
Model 2 + hsCRP	1.08 [0.99; 1.18], P = 0.073
Model 2 + leptin	1.08 [0.99; 1.18], P = 0.089
Model 2 + eGFR, insulin, hsCRP, leptin	1.07 [0.98; 1.17], P = 0.153

The associations were computed using Cox regression models per 1-SD increment of log (MR-proADM) and CT-proET-1. The distributions of MR-proADM, insulin, hsCRP, and leptin were right-skewed and thus, were log-transformed to approximate normality

*CI* confidence interval, *CT-proET-1* C-terminal-proendothelin-1, *eGFR* estimated glomerular filtration rate, *hsCRP* high-sensitivity C-reactive protein, *MR-proADM* mid-regional-proadrenomedullin

Model 1: adjusted for age (continuous, in years), sex (man/woman) and cohort (as a stratum variable);

Model 2: Model 1 + actual hypertension (yes/no), total and high-density lipoprotein cholesterol (continuous, in mmol/l), current smoking status (yes/no) and body mass index (continuous, in kg/m^2^)

^a^97% of study participants were fasting at least 4 h and the exclusion of those who were not fasting or whose fasting status was unknown did not change the results

We observed significant interactions of MR-proADM with BMI and waist circumference with respect to the association with incident type 2 diabetes (FDR < 0.05) (Table 3). When stratified by BMI, the positive association between MR-proADM and incident type 2 diabetes was only significant in obese participants. The HRs [95%CIs] per 1-SD increase of log MR-proADM were 1.19 [1.05; 1.34] in obese and 1.02 [0.90; 1.15] in non-obese participants. The results were similar when we stratified by waist circumference (Table 3). In an analysis where we further adjusted for eGFR, insulin, hsCRP and leptin, the association between MR-proADM and incident type 2 diabetes was attenuated, but remained significant in obese participants (HRs [95% CIs] per 1-SD increase of log MR-proADM: 1.14 [1.01; 1.29] in obese and 1.00 [0.88; 1.13] in non-obese participants). No significant differences could be detected in the association between MR-proADM and incident type 2 diabetes across sex and hypertension categories (Table 3).

**Table 3:** Subgroup analysis of the association of CT-proET-1 and MR-proADM with incident type 2 diabetes

	N cases/PY	CT-proET-1		MR-proADM	
		Hazard ratio [95%CI]	*P*-interaction	Hazard ratio [95%CI]	*P*-interaction
Overall	862/149,937	1.10 [1.03; 1.18], P = 0.008		1.11 [1.02; 1.21], P = 0.016	
BMI (kg/m^2^)			0.020		< 0.001 ^a^
Obese (≥ 30)	420/22,897	1.09 [0.99; 1.20], P = 0.070		1.19 [1.05; 1.34], P = 0.005	
Non-obese (< 30)	442/127,040	1.11 [1.00; 1.24], P = 0.058		1.02 [0.90; 1.15], P = 0.741	
Waist circumference (cm)			0.348		0.001 ^a^
Obese (Men: ≥ 102, Women: ≥ 88)	470/30,126	1.09 [1.00; 1.20], P = 0.055		1.15 [1.03; 1.28], P = 0.013	
Non-obese (Men: < 102, Women: < 88)	392/119,811	1.10 [0.98; 1.24], P = 0.116		1.01 [0.88; 1.15], P = 0.909	
Sex			0.145		0.157
Men	615/91,156	1.07 [0.97; 1.17], P = 0.164		1.06 [0.96; 1.19], P = 0.257	
Women	247/58,781	1.19 [1.05; 1.35], P = 0.006		1.25 [1.08; 1.46], P = 0.004	
Actual hypertension^b^			0.161		0.374
Yes	608/60,423	1.10 [1.01; 1.19], P = 0.026		1.12 [1.02; 1.24], P = 0.023	
No	254/89,513	1.16 [0.99; 1.35], P = 0.069		1.10 [0.93; 1.30], P = 0.267	

The associations were computed using Cox regression models per 1-SD increment of log (MR-proADM) and CT-proET-1

The models included study cohort as a stratum variable and were adjusted for age (continuous, in years), sex (men/women), actual hypertension (yes/no), total and HDL cholesterol (continuous, in mmol/l), current smoking status (yes/no) and BMI (continuous, in kg/m^2^) (waist circumference (continuous, in cm) instead of BMI in models for waist circumference)

Actual hypertension was defined as having systolic blood pressure ≥ 140 mmHg, diastolic blood pressure ≥ 90 mmHg or using antihypertensive medication

*BMI* body mass index, *CI* confidence interval, *CT-proET-1* C-terminal-proendothelin-1, *MR-proADM* mid-regional-proadrenomedullin, *PY* person-years

^a^Remained significant (FDR < 0.05) after correcting for multiple testing with the Benjamini–Hochberg method

^b^Actual hypertension was defined as systolic blood pressure ≥ 140 mmHg, diastolic blood pressure ≥ 90 mmHg or using antihypertensive medication

For CT-proET-1, no relevant interactions with BMI, waist circumference, sex and hypertension were observed with respect to incident type 2 diabetes under FDR < 0.05 (Table 3). The distribution of CT-proET-1 and MR-proADM by subgroup are presented in Additional file 1: Fig. S5.

### Mendelian randomisation analysis

We identified one SNP that is specific for CT-proET-1 in the *EDN-1* gene (rs5370) and one SNP that is specific for MR-proADM in the *ADM* gene (rs2957692) and included them as the genetic IVs. The genetic associations with each vasoactive peptide and with type 2 diabetes were extracted from the previously mentioned GWA studies [27–29].

In line with the findings from the time-to-event analysis, our Mendelian randomisation analysis showed a significant positive association between genetically predicted CT-proET-1 and type 2 diabetes risk. The OR [95% CI] was 1.12 [1.03; 1.22]. Conversely, we did not observe a significant association between genetically predicted MR-proADM and type 2 diabetes risk. The OR [95%CI] for MR-proADM was 0.97 [0.74; 1.27]. Sensitivity analyses using the likelihood-based method yielded similar results (OR [95%CI]: 1.12 [1.02; 1.22] for CT-proET-1 and 0.96 [0.73; 1.27] for MR-proADM) (Table 4).

**Table 4:** Results for the two-sample Mendelian randomisation analysis

SNP (Gene)	Effect allele	Phenotype	Association estimates with vasoactive peptides per 1-SD difference		Association estimates with type 2 diabetes		Methods	Mendelian randomisation estimates on odds ratio scale [95% CI]
			β (SE)^a^	P-value	β (SE)	P-value		
rs5370 (*EDN1*)	T	CT-proET-1	0.213 (0.020)	1.49E−27	0.024 (0.009)	0.002	Wald ratio	1.12 [1.03; 1.22]; P = 0.011
							Maximum likelihood	1.12 [1.02; 1.22]; P = 0.013
rs2957692 (*ADM*)	G	MR-proADM	− 0.115 (0.015)	1.05E−12	0.004 (0.016)	0.798	Wald ratio	0.97 [0.74: 1.27]; P = 0.798
							Maximum likelihood	0.96 [0.73; 1.27]; P = 0.798

^a^Standardized β estimates

*CI* confidence interval, *CT-proET-1* C-terminal-proendothelin-1, *MR-proADM* mid-regional-proadrenomedullin, *SE* standard error, *SNP* single nucleotide polymorphism

## Discussion

In the current study, we observed that higher concentrations of both CT-proET-1 and MR-proADM were significantly associated with a higher incidence of type 2 diabetes. This is the first study to demonstrate a positive association between MR-proADM and incident type 2 diabetes independently of classical diabetes risk factors and that the association was more apparent in obese than in non-obese individuals. Using Mendelian randomisation approaches, we added further evidence that genetically predicted CT-proET-1 was significantly associated with a higher risk of type 2 diabetes. No significant association between genetically predicted MR-proADM and type 2 diabetes risk was documented.

Our results corroborate the few existing prospective analyses reporting a positive association of CT-proET-1 with incident type 2 diabetes [21, 22] and added evidence for a similar association for MR-proADM, particularly in obese individuals. In a previous study using data from 7953 participants of the Prevention of Vascular and Renal End-stage Disease Cohort [18], the authors also reported a significant positive association between MR-proADM and incident type 2 diabetes in a model adjusted for age and sex. However, the association was no longer significant in a multivariable model adjusted for classical diabetes risk factors. Compared with the previous prospective studies, our study represents the largest population-based cohort study examining the association of CT-proET-1 and MR-proADM with incident type 2 diabetes.

Factors underlying the association between CT-proET-1 and incident type 2 diabetes are not well understood. Studies conducted thus far have demonstrated that overexpression of ET-1 directly limits insulin actions. In skeletal muscles, the activation of endothelin receptor type-A by ET-1 suppresses insulin-mediated Akt phosphorylation and reduces glucose uptake [5, 31]. ET-1 also disrupts insulin-regulated glucose transporter 4 translocation to the plasma membrane [32]. In adipocytes, ET-1 blocks free fatty acid uptake and induces lipolysis, resulting in increased free fatty acid concentrations [33, 34]. Moreover, the interplay between increased free fatty acids and impaired glucose uptake may further exacerbate the dysregulation of lipid metabolism and energy homeostasis in insulin-resistant states [33]. Conversely, the inhibition of ET-1 signalling improves insulin sensitivity [35, 36]. Altogether, these biological effects suggest that ET-1 promotes insulin resistance and impaired glucose tolerance and thereby increases the risk of type 2 diabetes.

Furthermore, ET-1 is a potent vasoconstrictor, which plays an important role in the pathogenesis of hypertension and chronic kidney disease [3, 37], both are known to be associated with type 2 diabetes. ET-1 signalling also has been linked to increased leptin production [38] and stimulates the secretion of pro-inflammatory cytokines known to be involved in the development of metabolic disorders [6, 39]. However, in the present study, the positive association between CT-proET-1 and incident type 2 diabetes remained stable after additional adjustment for eGFR, insulin, hsCRP, leptin, and fasting glucose suggesting other possible explanations.

With regard to ADM, the underlying mechanisms linking higher concentrations with an increased risk of type 2 diabetes seem to be less straightforward. Evidence from in vivo and in vitro studies suggest that ADM could counteract insulin resistance through its antioxidant effects and the inhibition of insulin secretion [9, 11]. The latter notion also implicates ADM in maintaining insulin homeostasis [11]. ADM also has anti-inflammatory actions [40]. In obesity, ADM expression is upregulated in adipocytes and circulating ADM concentrations are increased [10]. Evidence from previous epidemiological studies also suggest positive associations of ADM with BMI and waist circumference [41, 42]. Factors that upregulate ADM production in obesity are incompletely understood. In an experimental study using a euglycaemic-hyperinsulinemic clamp technique, acute hyperinsulinemia was demonstrated to induce circulating ADM concentrations in obese, but not in lean individuals [43]. This evidence could explain the more apparent association of MR-proADM with incident type 2 diabetes in obese than in non-obese individuals seen in the current study. Furthermore, oxidative stress, insulin resistance, low-grade inflammation and dyslipidaemia, conditions that are commonly found in obesity, were also associated with increased MR-proADM concentrations [16, 44]. The increased ADM release in adipocytes seems to be a compensatory action attempting to restrain insulin homeostasis rather than a causal factor of insulin resistance thus, type 2 diabetes. Of note, in our data, the association between MR-proADM and incident type 2 diabetes was attenuated when we further controlled for insulin, hsCRP and leptin. We also did not observe a significant association between genetically predicted MR-proADM and type 2 diabetes risk in our Mendelian randomisation analysis. However, a non-significant association is not evidence for no association. Further studies are needed to confirm our findings.

Our study has some limitations that should be considered. As only single measurements of CT-proET-1 and MR-proADM were available at baseline we could not take into account the intra-individual variation. This could have led to exposure misclassification and regression dilution bias. In the current study, the harmonized data on several known diabetes risk factors, such as physical activity, diet and family history of diabetes, were lacking, which could have led to some degree of residual confounding. Our study participants were predominantly of European descent, which means that further studies need to confirm our findings in other ethnic groups. Finally, due to a very limited number of genetic IVs, we were unable to perform more robust analyses for our Mendelian randomisation.

Our study also has several strengths including the prospective, population-based design and the long-term follow-up with a median of 13.8 years. The use of harmonized data from the population-based cohorts participating in the BiomarCaRE project allows us to include a large sample size. To our knowledge, our study represents the so far largest population-based cohort study examining the association of CT-proET-1 and MR-proADM with incident type 2 diabetes. Furthermore, standardized epidemiological and laboratory procedures based on individual level data also allow for the best possible data analyses, including thorough adjustments for different diabetes risk factors and subgroup analyses.

## Conclusions

In conclusion, higher concentrations of CT-proET-1 and MR-proADM were associated with incident type 2 diabetes. However, the positive association between MR-proADM and incident type 2 diabetes seemed to be modified by body composition, with a more apparent association in obese than in non-obese individuals. Our Mendelian randomisation analysis further suggests a probable causal link between CT-proET-1 and type 2 diabetes. These findings raise the possibility that ET-1 might be implicated in the pathogenesis of type 2 diabetes. Future studies are warranted to examine the utility of both peptides in risk stratification of type 2 diabetes for a better health care decision and their potential as targets for antidiabetic therapy.

## Supplementary Information

**Additional file 1: Table S1.** Overview of contributing BiomarCaRE cohorts. **Table S2.** Intra-assay and inter-assay coefficients of variation for CT-proET-1 and MR-proADM by participating BiomarCaRE cohort. **Table S3.** Characteristics of participants with complete data. **Table S4.** Characteristics of participant by participating cohort. **Table S5.** Association of CT-proET-1 and MR-proADM with incident type 2 diabetes additionally controlling for baseline fasting glucose in subgroup with available fasting glucose measurements. **Figure S1.** Flowchart showing sample size and reasons for exclusion. **Figure S2.** The distribution (frequency histogram) of CT-proET-1 (A) and MR-proADM (B) in the study population. **Figure S3.** Association between CT-proET-1 and incident type 2 diabetes in each participating BiomarCaRE cohort. **Figure S4.** Association between MR-proADM and incident type 2 diabetes in each participating BiomarCaRE cohort. **Figure S5.** The distribution of CT-proET-1 and MR-proADM by subgroup. **Text S1.** Laboratory measurements for other biomarkers used in the analyses. **Text S2.** Procedure for the univariate Mendelian randomisation analysis.

## Data Availability

Data are not made available in a public repository. Access to the data is restricted by the ethical approvals and the legislation of the European Union. Approval by the Principal Investigator of each participating cohort study and the MORGAM/ BiomarCaRE Steering Group are required for release of the data. The MORGAM Manual at https://www.thl.f/publications/morgam/manual/contents.htm gives more information on access to the data.

## Abbreviations

ADM: Adrenomedullin
BiomarCaRE: Biomarkers for Cardiovascular Risk Assessment in Europe
BMI: Body mass index
CT-proET-1: C-terminal-proendothelin-1
CI: Confidence interval
eGFR: Estimated glomerular filtration rate
GWA: Genome-wide association
ET-1: Endothelin-1
FDR: False discovery rate
HDL: High density lipoprotein
HR: Hazard ratio
hsCRP: High-sensitivity C-reactive protein
IV: Instrumental variable
KORA: Cooperative Health Research in the Region of Augsburg
LOD: Limit of detection
MICE: Multiple imputation by chained equations
MONICA: Multinational Monitoring of Trends and Determinants in Cardiovascular Disease
MORGAM: MONICA Risk, Genetics, Archiving and Monograph
MR-proADM: Mid-regional-proadrenomedullin
OR: Odds ratio
PH: Proportional hazard
PRIME: Prospective Epidemiological Study of Myocardial Infarction
SD: Standard deviation
SNP: Single nucleotide polymorphism
