# Supplementary material for: Associations of the vasoactive peptides CT-proET-1 and MR-proADM with incident type 2 diabetes: results from the BiomarCaRE Consortium
Source: Cardiovasc Diabetol. 2022 Jun 9;21:99. doi: 10.1186/s12933-022-01513-9 (PMC9185875; doi:10.1186/s12933-022-01513-9)
Supplement: Supplementary file 1 — Additional file 1: Table S1. Overview of contributing BiomarCaRE cohorts. Table S2. Intra-assay and inter-assay coefficients of variation for CT-proET-1 and MR-proADM by participating BiomarCaRE cohort. Table S3. Characteristics of participants with complete data. Table S4. Characteristics of participant by participating cohort. Table S5. Association of CT-proET-1 and MR-proADM with incident type 2 diabetes additionally controlling for baseline fasting glucose in subgroup with available fasting glucose measurements. Figure S1. Flowchart showing sample size and reasons for exclusion. Figure S2. The distribution (frequency histogram) of CT-proET-1 (A) and MR-proADM (B) in the study population. Figure S3. Association between CT-proET-1 and incident type 2 diabetes in each participating BiomarCaRE cohort. Figure S4. Association between MR-proADM and incident type 2 diabetes in each participating BiomarCaRE cohort. Figure S5. The distribution of CT-proET-1 and MR-proADM by subgroup. Text S1. Laboratory measurements for other biomarkers used in the analyses. Text S2. Procedure for the univariate Mendelian randomisation analysis. [file 12933_2022_1513_MOESM1_ESM.docx]

**Additional File 1**

**Associations of the Vasoactive Peptides CT-proET-1 and MR-proADM with Incident Type 2 Diabetes: Results from the BiomarCaRE Consortium**

Chaterina Sujana^1,2,3^, Veikko Salomaa^4^, Frank Kee^5^, Jochen Seissler^6^, Pekka Jousilahti^4^, Charlotte Neville^5^, Cornelia Then^6^, Wolfgang Koenig^7,8,9^, Kari Kuulasmaa^4^, Jaakko Reinikainen^4^, Stefan Blankenberg^10,11^, Tanja Zeller^10,11^, Christian Herder^12,13,14^, Ulrich Mansmann^2^, Annette Peters^1,3,9^, Barbara Thorand^1,3^, for the BiomarCaRE Consortium

^1^ Institute of Epidemiology, Helmholtz Zentrum München - German Research Center for Environmental Health, Neuherberg, Germany;

^2^ Institute for Medical Information Processing, Biometry, and Epidemiology (IBE), Pettenkofer School of Public Health, Ludwig-Maximilians-Universität München, Munich, Germany;

^3^ German Center for Diabetes Research (DZD), Partner München-Neuherberg, Neuherberg, Germany.

^4^ Department of Public Health and Welfare, Finnish Institute for Health and Welfare, Helsinki, Finland;

^5^ Centre for Public Health, Queens University of Belfast, Belfast, Northern Ireland, UK;

^6^ Diabetes Zentrum, Medizinische Klinik und Poliklinik IV, Klinikum der Ludwig-Maximilians-Universität München, Munich, Germany;

^7^ Institute of Epidemiology and Medical Biometry - University of Ulm, Ulm, Germany;

^8^ Deutsches Herzzentrum München, Technische Universität München, Munich, Germany;

^9^ German Centre for Cardiovascular Research (DZHK e.V.), partner site Munich Heart Alliance, Munich, Germany;

^10^ Department for General and Interventional Cardiology, University Heart Center Hamburg, Hamburg, Germany;

^11^ German Centre for Cardiovascular Research (DZHK e.V.), partner site Hamburg, Lübeck, Kiel, Hamburg, Germany;

^12^ Institute for Clinical Diabetology, German Diabetes Center, Leibniz Center for Diabetes Research at Heinrich-Heine-University Düsseldorf, Düsseldorf, Germany;

^13^ Division of Endocrinology and Diabetology, Medical Faculty and University Hospital Düsseldorf, Heinrich-Heine-University Düsseldorf, Düsseldorf, Germany;

^14^ German Center for Diabetes Research (DZD), Partner Düsseldorf, Neuherberg, Germany.

# **Table S1. Overview of contributing BiomarCaRE cohorts**

| **Cohort** | **Country** | **Short description** |
| --- | --- | --- |
| Kooperative Gesundheits-forschung in der Region Augsburg (KORA) F4 | Germany | The Cooperative Health Research in the Region of Augsburg F4 (KORA F4; 2006-2008) is the first follow-up examination of the fourth survey of the population-based KORA study (KORA S4; 1999–2001). The S4 study comprises randomly selected respondents aged 25-74 years from the city of Augsburg in Bavaria, Southern Germany and its two surrounding counties. List of municipalities and population registers were used as sampling frames. Out of 4,261 S4 participants, 3,080 participated in F4. The BiomarCaRE project includes 3,060 participants from F4. |
|  |  | Follow-up examinations of KORA S4 participants were conducted in 2006-2008 (F4) and 2013/2014 (FF4). Follow-up questionnaires were sent to all KORA S4 study participants in 2008/2009 and 2016 to obtain information on the occurrence of chronic diseases and risk factors. The core BiomarCaRE database includes follow-up data until 2009. |
|  |  | In the current analysis, we included data from the KORA F4 study who were followed for incident type 2 diabetes up to 2016. Prevalent or incident type 2 diabetes was defined as self-reported clinically diagnosed diabetes that could be validated by the responsible physician or hospital discharge letters, or by self-reported use of glucose-lowering medication. Furthermore, participants with clinical diagnoses or comorbidity ICD-code (ICD-9: 250) on the death certificate were coded as prevalent or incident diabetes. For incident diabetes cases, the self-reported date of diagnosis was assessed and generally verified by contacting the treating physician or medical chart review. When information on the type of diabetes was not available, it was considered to be type 2 diabetes if the age of the person was above 35 years at the time of diagnosis. |
|  |  | <https://www.thl.fi/publications/morgam/cohorts/full/germany/ger-auga.htm> |
| FINRISK | Finland | The FINRISK study is a series of population-based cardiovascular risk factor surveys carried out every five years in five (or six in 2002) districts of Finland, including North Karelia, Northern Savo (former Kuopio), South-western Finland, Oulu Province, Lapland province (in 2002 only) and the region of Helsinki and Vantaa. A stratified random sample was drawn for each survey from the national population register; the age-range was 25-74 years. All individuals enrolled in the study received a physical examination, a self-administered questionnaire, and a blood sample examination. |
|  |  | In 1997, altogether 11,500 individuals were invited and 8,444 (73%) participated in the clinical examination. During the follow-up time the National Hospital Discharge Register, the National Causes of Death Register and the National Drug Reimbursement Register were used to identify endpoints. Participants were followed up until December 31^st^, 2010. |
|  |  | The cohorts were linked to the Hospital Discharge Register and Causes of Death Register and drug reimbursement registers. A hospitalization or death with the ICD-8/9 code 250 or with any of the ICD-10 codes E10, E11 and E14 was considered to indicate diabetes. Likewise, the appearance of “special reimbursements” for diabetes mellitus, i.e., KELA code 103, or purchases of drugs with the ATC code A10* were taken as diabetes. The type of diabetes was then determined as follows: If the age of the patient was < 35 at the time of diagnosis and the treatment was insulin only, the person was considered to have type 1 diabetes. All others were considered to have type 2 diabetes. |
|  |  | <http://www.thl.fi/publications/morgam/cohorts/full/finland/fin-fina.htm> |
| Prospective Epidemiological Study of Myocardial Infarction | United Kingdom | The PRIME study examined the classic and putative cardiovascular risk factors to explain the large difference in heart disease incidence between Ireland and France. The study includes four cohorts of men aged 50-59; from Belfast, Northern Ireland (N= 2,745) and Lille (N= 2,633), Toulouse (N= 2,610) and Strasbourg (N= 2,612) in France. |
| (PRIME) Belfast |  | The current study only includes the Belfast cohort, since data on vasoactive peptides of interest was not available for the French cohorts. Baseline examinations took place in 1991-1994 and targeted cohorts that had broadly similar social class structures to the background population. Study participants were followed up until 2012 through annual follow up questionnaires with verification against national death registers, medical records, and hospital discharge diagnoses. Endpoints were validated by expert medical committee. |
|  |  | Type 2 diabetes cases identified by contacting the practitioner of each subject who self-reported type 2 diabetes to validate diabetes type, treatment and diagnosis date. |
|  |  | <http://www.thl.fi/publications/morgam/cohorts/full/uk/unk-bela.htm> |

| **Cohort** | **CT-proET-1** | **MR-proADM** |
| --- | --- | --- |

# **Table S2. Intra-assay and inter-assay coefficients of variation for CT-proET-1 and MR-proADM by participating BiomarCaRE cohort**

|  | **Intra-assay (%)** | **Inter-assay (%)** | **Intra-assay (%)** | **Inter-assay (%)** |
| --- | --- | --- | --- | --- |
| FINRISK | 2.16 - 2.61 | 1.74 - 8.79 | 2.17 | 2.43 |
| PRIME Belfast | 2.61 | 3.57 | 2.17 | 2.43 |
| KORA F4 | 4.8 | 6.9 | 4.5 | 7.8 |

Abbreviations: CT-proET-1, C-terminal-proendothelin-1; KORA, Cooperative Health Research in the Region of Augsburg Study; MR-proADM, mid-regional-proadrenomedullin; PRIME, Prospective Epidemiological Study of Myocardial Infarction.

# **Table S3. Characteristics of participants with complete data**

|  | **values** | **% missing** |
| --- | --- | --- |
| Number of individuals | 12,006 |  |
| Event (N (%)) |  | 0% |
| Incident type 2 diabetes | 862 (7.2%) |  |
| Censored | 11,144 (92.8%) |  |
| Cohort (N (%)) |  | 0% |
| FINRISK | 7,336 (61.1%) |  |
| PRIME Belfast | 2,496 (20.8%) |  |
| KORA F4 | 2,174 (18.1%) |  |
| CT-proET-1, in pmol/l (mean (SD)) | 51.3 (13.5) | 17.9% |
| MR-proADM, in nmol/l (geometric mean (antilog SD)) | 0.45 (1.31) | 17.9% |
| Age, in years (mean (SD)) | 49.4 (11.8) | 0% |
| Male (N (%)) | 7,072 (58.9%) | 0% |
| Body mass index, in kg/m^2^ (mean (SD)) | 26.5 (4.25) | 0.1% |
| Actual hypertension ^a^ (N (%)) | 4,886 (41.0%) | 0.7% |
| Systolic blood pressure, in mmHg (mean (SD)) | 132.1 (20.1) | 0.1% |
| Diastolic blood pressure, in mmHg (mean (SD)) | 81.0 (11.3) | 0.1% |
| Use of antihypertensive medication (N (%)) | 1,294 (11.0%) | 1.6% |
| Current smoker (N (%)) | 3,234 (26.9%) | 0% |
| Total cholesterol, in mmol/l (mean (SD)) | 5.59 (1.05) | 0.4% |
| HDL, in mmol/l (mean (SD)) | 1.38 (0.37) | 0.4% |
| eGFR (ml/min/1.73m^2^) (mean (SD)) | 89.5 (19.1) | 10.5% |
| hsCRP (mg/l) (geometric mean (antilog SD)) | 1.22 (3.03) | 5.7% |
| Insulin (microU/ml) (geometric mean (antilog SD)) | 5.81 (1.84) | 8.1% |
| Leptin (ng/ml) (geometric mean (antilog SD)) | 7.37 (2.69) | 6.1% |
| Fasting glucose (mmol/l) (geometric mean (antilog SD)) ^b^ | 5.03 (1.13) | 27.0% |

Data are presented as frequency (percentage) for categorical variables and as mean (SD) for continuous variables. Continuous variables with skewed distributions are presented as geometric mean (antilog SD). ^a^ Actual hypertension was defined as having systolic blood pressure ≥ 140 mmHg, diastolic blood pressure ≥ 90 mmHg or using antihypertensive medication. ^b^ Data were available and calculated in participants of FINRISK and KORA F4 who fasted at least 4 hours.

Abbreviations: eGFR, estimated glomerular filtration rate; CT-proET-1, C-terminal-proendothelin-1; HDL, high-density lipoprotein; hsCRP, high-sensitivity C-reactive protein; KORA, Cooperative Health Research in the Region of Augsburg Study; MR-proADM, mid-regional-proadrenomedullin; PRIME, Prospective Epidemiological Study of Myocardial Infarction; SD, standard deviation.

# **Table S4. Characteristics of participant by participating cohort**

|  | **FINRISK** | **PRIME Belfast** | **KORA F4** |
| --- | --- | --- | --- |
| N | 7,336 | 2,496 | 2,174 |
| Examination years | 1997 | 1991-1994 | 2006-2008 |
| Median of follow-up time in years (IQR) | 13.8 (0.11) | 18.0 (4.38) | 8.1 (1.02) |
| Incident type 2 diabetes (N (%)) | 531 (7.2) | 240 (9.6) | 91 (4.2) |
| Person-years | 95,093 | 38,380 | 16,464 |
| Incidence rate of type 2 diabetes per 1000 person-years (95% CI) | 5.58 (5.12; 6.08) | 6.25 (5.49; 7.10) | 5.53 (4.45; 6.79) |
| CT-proET-1, in pmol/l (mean (SD)) | 53.2 (14.5) | 49.2 (9.7) | 44.2 (10.5) |
| MR-proADM, in nmol/l (geometric mean (antilog SD)) | 0.46 (1.34) | 0.44 (1.23) | 0.47 (1.26) |
| Age, in years (mean (SD)) | 47.0 (13.1) | 54.7 (2.90) | 51.5 (10.7) |
| Male (N (%)) | 3,569 (48.7) | 2,496 (100.0) | 1,007 (46.3) |
| Body mass index, in kg/m^2^ (mean (SD)) | 26.4 (4.42) | 26.2 (3.38) | 26.9 (4.55) |
| Waist circumference, in cm (mean (SD)) | 87.4 (13.3) | 91.1 (9.3) | 91.2 (13.6) |
| Actual hypertension (N (%)) ^a^ | 3,273 (44.6) | 1,044 (41.8) | 582 (26.8) |
| Systolic blood pressure, in mmHg (mean (SD)) | 134.9 (19.7) | 133.7 (20.3) | 120.8 (17.5) |
| Diastolic blood pressure, in mmHg (mean (SD)) | 82.1 (11.3) | 81.8 (11.4) | 76.1 (9.9) |
| Use of antihypertensive medication (N (%)) | 748 (10.2) | 217 (8.7) | 343 (15.8) |
| Current smoker (N (%)) | 1,991 (27.1) | 786 (31.5) | 457 (21.0) |
| Total cholesterol, in mmol/l (mean (SD)) | 5.49 (1.06) | 5.88 (1.02) | 5.58 (1.00) |
| HDL, in mmol/l (mean (SD)) | 1.41 (0.36) | 1.19 (0.32) | 1.47 (0.38) |
| eGFR (ml/min/1.73m^2^) (mean (SD)) | 89.7 (19.9) | 83.9 (20.9) | 92.8 (14.2) |
| hsCRP (mg/l) (geometric mean (antilog SD)) | 1.16 (3.04) | 1.63 (2.87) | 1.09 (3.06) |
| Insulin (microU/ml) (geometric mean (antilog SD)) | 5.18 (1.87) | 5.77 (1.70) | 8.48 (1.68) |
| Leptin (ng/ml) (geometric mean (antilog SD)) | 7.98 (2.57) | 4.32 (2.10) | 9.85 (3.20) |
| Fasting glucose (mmol/l) (geometric mean (antilog SD)) ^b^ | 4.96 (1.14) | NA | 5.17 (1.11) |

Data are presented as frequency (percentage) for categorical variables and as mean (SD) for continuous variables. Continuous variables with skewed distributions are presented as geometric mean (antilog SD). ^a^ Actual hypertension was defined as having systolic blood pressure ≥ 140 mmHg, diastolic blood pressure ≥ 90 mmHg or using antihypertensive medication. ^b^ Data were available and calculated in 6,952 FINRISK participants and 2,160 KORA F4 participants who fasted at least 4 hours.

Abbreviations: eGFR, estimated glomerular filtration rate; CT-proET-1, C-terminal-proendothelin-1; HDL, high-density lipoprotein; hsCRP, high-sensitivity C-reactive protein; KORA, Cooperative Health Research in the Region of Augsburg Study; MR-proADM, mid-regional-proadrenomedullin; PRIME, Prospective Epidemiological Study of Myocardial Infarction; SD, standard deviation.

**Table S5. Association of CT-proET-1 and MR-proADM with incident type 2 diabetes additionally controlling for baseline fasting glucose in subgroup with available fasting glucose measurements**

|  | **Adjustment** | **Hazard ratio [95%CI]** |
| --- | --- | --- |
|  |  | **N cases / person-years = 593 / 106,651** |
| CT-proET-1 | Model 1 | 1.32 [1.23; 1.43], P < 0.001 |
|  | Model 2 | 1.13 [1.04; 1.22], P = 0.004 |
|  | Model 2 + eGFR | 1.13 [1.04; 1.23], P = 0.003 |
|  | Model 2 + fasting insulin | 1.12 [1.03; 1.22], P = 0.006 |
|  | Model 2 + hsCRP | 1.11 [1.02; 1.20], P = 0.012 |
|  | Model 2 + leptin | 1.12 [1.04; 1.22], P = 0.005 |
|  | Model 2 + fasting glucose | 1.17 [1.08; 1.27], P < 0.001 |
|  | Model 2 + eGFR, fasting insulin, hsCRP, leptin, fasting glucose | 1.16 [1.07; 1.25], P = 0.001 |
| MR-proADM | Model 1 | 1.66 [1.51; 1.81], P < 0.001 |
|  | Model 2 | 1.15 [1.04; 1.28], P = 0.006 |
|  | Model 2 + eGFR | 1.16 [1.05; 1.29], P = 0.005 |
|  | Model 2 + fasting insulin | 1.12 [1.02; 1.24], P = 0.024 |
|  | Model 2 + hsCRP | 1.12 [1.01; 1.24], P = 0.033 |
|  | Model 2 + leptin | 1.14 [1.02; 1.26], P = 0.017 |
|  | Model 2 + fasting glucose | 1.23 [1.11; 1.36], P < 0.001 |
|  | Model 2 + eGFR, fasting insulin, hsCRP, leptin, fasting glucose | 1.19 [1.07; 1.32], P = 0.001 |

The associations were computed using Cox regression models per 1-SD increment of log (MR-proADM) and CT-proET-1. Data on fasting glucose were only available in FINRISK and KORA; thus, the analyses were performed in these cohorts (N= 9,112). The distributions of MR-proADM, insulin, hsCRP, leptin, and fasting glucose were right-skewed and thus, were log-transformed to approximate normality.

Model 1: adjusted for age (continuous, in years), sex (man/woman) and cohort (as a stratum variable);

Model 2: Model 1 + actual hypertension (yes/no), total and high-density lipoprotein cholesterol (continuous, in mmol/l), current smoking status (yes/no) and body mass index (continuous, in kg/m2).

Abbreviations: CI, confidence interval; CT-proET-1, C-terminal-proendothelin-1; eGFR, estimated glomerular filtration rate; hsCRP, high-sensitivity C-reactive protein; MR-proADM, mid-regional-proadrenomedullin.

excl.:

14,249 eligible participants

from three population-based cohorts of the Biomarkers for Cardiovascular Risk Assessment in Europe (BiomarCaRE) consortium

N= 12,006

included in the analysis

(862 incident cases of type 2 diabetes during follow-up)

- Individuals with prevalent diabetes and/or cardiovascular disease or insufficient data on diabetes and/or or cardiovascular disease status at baseline (N= 2,172)
- Individuals without follow-up data on type 2 diabetes

(N= 71)

excluded:

**Figure S1. Flowchart showing sample size and reasons for exclusion**


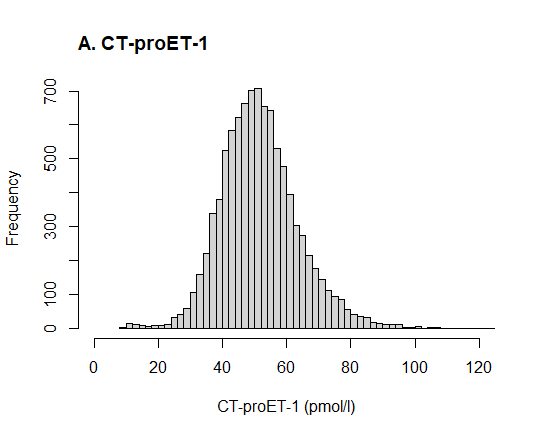

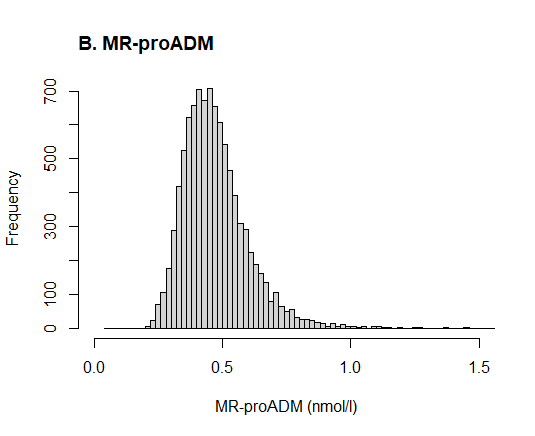


**Figure S2. The distribution of CT-proET-1 (A) and MR-proADM (B) in the study population.** The distributions of both vasoactive peptides were examined in all study participants with complete data for the main analysis. Abbreviations: CT-proET-1, C-terminal-proendothelin-1; MR-proADM, mid-regional-proadrenomedullin.


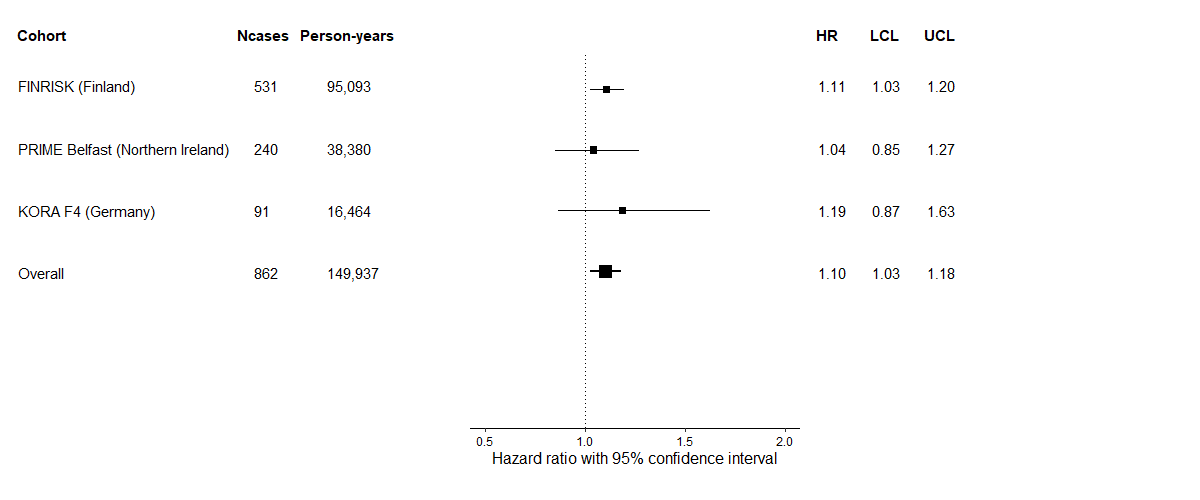


**Figure S3. Association between CT-proET-1 and incident type 2 diabetes in each participating BiomarCaRE cohort.** Hazard ratios for each study cohort were computed using Cox models. The models were adjusted for age (continuous, in years), sex (men/women), body mass index (continuous, in kg/m^2^), current smoking (yes/no), actual hypertension (yes/no), total and high-density lipoprotein cholesterol (continuous, in mmol/l). CT-proET-1 was (0,1)-standardized in the total study population to evaluate the hazard ratios per 1-standard deviation increase. Overall estimate was calculated using DerSimonian-Laird random-effects model. Heterogeneity: *P*-value for interaction between CT-proET-1 and study cohort = 0.682; *P*-value for Cochran’s Q = 0.761; I^2^ = 0%. Black squares represent hazard ratios and bars represent 95% confidence intervals per 1-standard deviation increment of CT-proET-1. Abbreviations: BiomarCaRE, Biomarkers for Cardiovascular Risk Assessment in Europe; CT-proET-1, C-terminal-proendothelin-1; HR, hazard ratio; KORA, Cooperative Health Research in the Region of Augsburg Study; LCL, lower confidence limit; Ncases, number of incident type 2 diabetes cases; PRIME, Prospective Epidemiological Study of Myocardial Infarction; UCL, upper confidence limit.


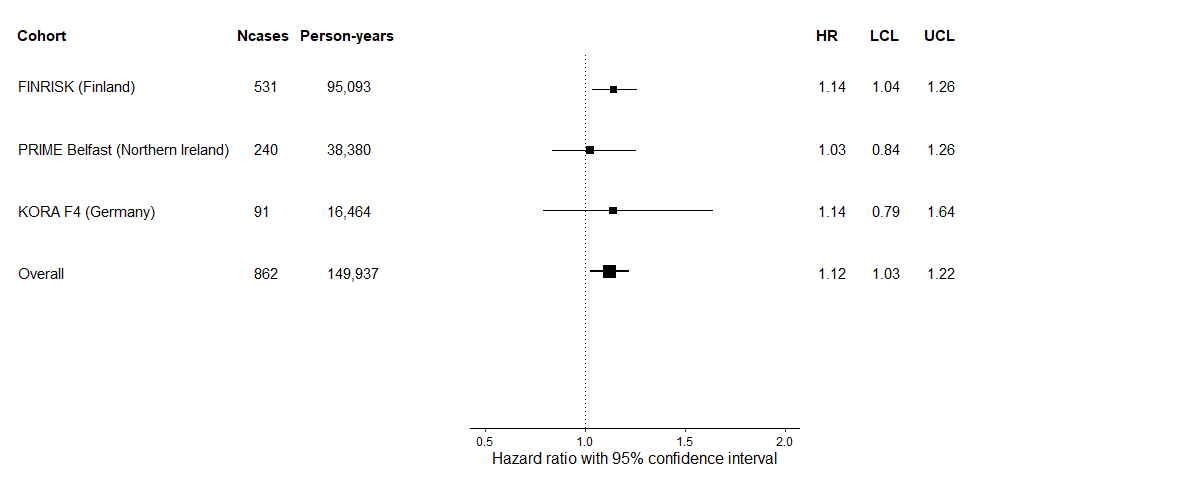


**Figure S4. Association between MR-proADM and incident type 2 diabetes in each participating BiomarCaRE cohort.** Hazard ratios for each study cohort were computed using Cox models. The models were adjusted for age (continuous, in years), sex (men/women), body mass index (continuous, in kg/m^2^), current smoking (yes/no), actual hypertension (yes/no), total and high-density lipoprotein cholesterol (continuous, in mmol/l). MR-proADM was log-transformed and (0,1)-standardized in the total study population to approximate normality and to evaluate the hazard ratios per 1-standard deviation increase. Overall estimate was calculated using DerSimonian-Laird random-effects model. Heterogeneity: *P*-value for interaction between MR-proADM and study cohort = 0.379; *P*-value for Cochran’s Q = 0.639; I^2^ = 0%. Black squares represent hazard ratios and bars represent 95% confidence intervals per 1-standard deviation increment of log MR-proADM. Abbreviations: BiomarCaRE, Biomarkers for Cardiovascular Risk Assessment in Europe; HR, hazard ratio; KORA, Cooperative Health Research in the Region of Augsburg Study; LCL, lower confidence limit; MR-proADM, mid-regional-proadrenomedullin; Ncases, number of incident type 2 diabetes cases; PRIME, Prospective Epidemiological Study of Myocardial Infarction; UCL, upper confidence limit.

| **Subgroup** | **CT-proET-1** | **MR-proADM** |
| --- | --- | --- |
| **BMI (kg/m^2^)** |  |  |
| Obese (≥ 30) vs  Non-obese (< 30) | 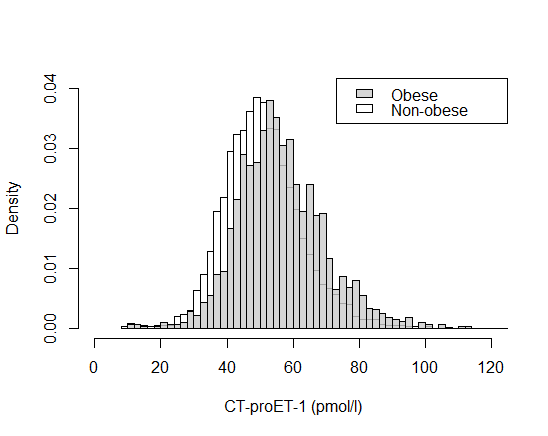 | 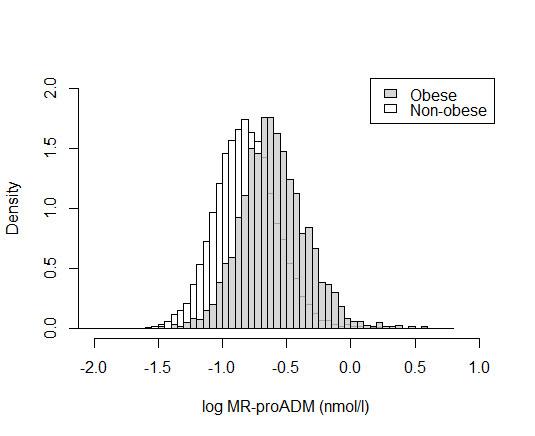 |
| **Waist circumference (cm)** |  |  |
| Obese (Men: ≥ 102, Women: ≥ 88) vs  Non-obese (Men: < 102, Women: < 88) | 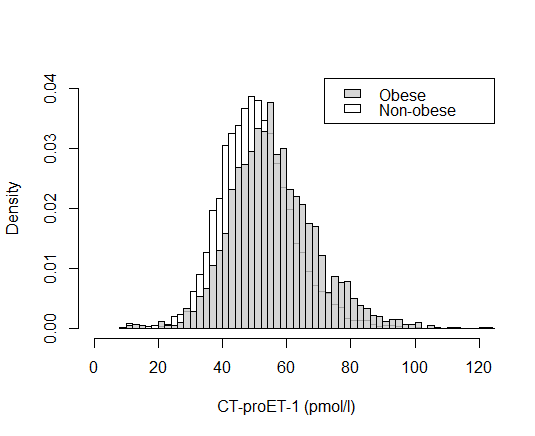 | 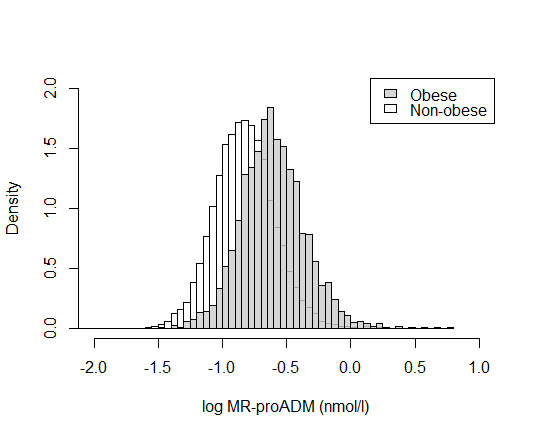 |
| **Sex** |  |  |
| Men vs Women | 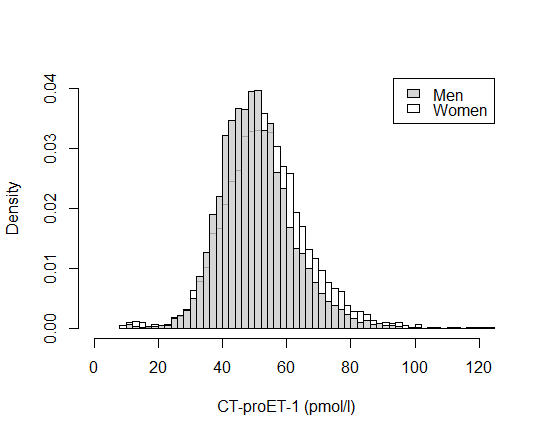 | 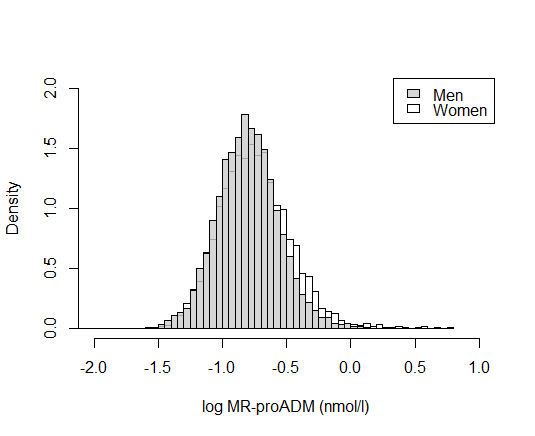 |
| **Actual hypertension^a^** |  |  |
| Hypertensive vs  Non-hypertensive | 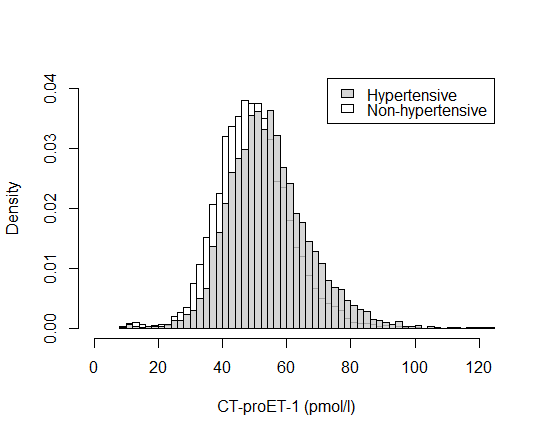 | 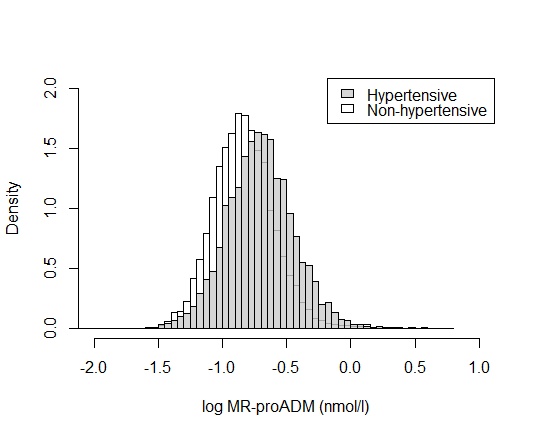 |

**Figure S5. The distribution of CT-proET-1 and MR-proADM by subgroup.** The distributions of both vasoactive peptides were examined in all study participants with complete data for the main analysis. ^a^Actual hypertension was defined as systolic blood pressure ≥ 140 mmHg, diastolic blood pressure ≥ 90 mmHg or using antihypertensive medication. Abbreviations: BMI, body mass index; CT-proET-1, C-terminal-proendothelin-1; MR-proADM, mid-regional-proadrenomedullin.

**Text S1. Laboratory measurements for other biomarkers used in the analyses**

Baseline concentrations of high-sensitivity C-reactive protein (hsCRP) were centrally measured in MORGAM/BiomarCaRE core laboratory for the FINRISK Study and the Prospective Epidemiological Study of Myocardial Infarction (PRIME) Belfast Study. hsCRP was measured from blood serum using latex immunoassay CRP16 (Turbidimetric / Immunoturbidimetric, Architect c8000 Abbott, Wiesbaden, Germany). In the Cooperative Health Research in the Augsburg Region Study (KORA) F4 Study, hsCRP was measured from plasma using a latex-enhanced immunonephelometry (BN II, Siemens, Erlangen, Germany).

Baseline concentrations of insulin were measured from serum using chemiluminescent microparticle immunoassay CMIA (Abbott, Architect i2000) for FINRISK and PRIME Belfast. In PRIME Belfast, data were measured after at least 8 hours fasting. In FINRISK, vast majority of the participants fasted at least 4 hours. Around 5% of FINRISK study participants did not fast or for whom the fasting status was unknown. In KORA F4, the data were measured from serum after an overnight fasting of at least 8 hours using electrochemiluminescence immunoassay (ECLIA, Roche Diagnostics GmbH, Mannheim, Germany) on the Cobas e602 instrument.

Baseline concentrations of leptin were measured from serum with an enzyme immunoassay technique using the Quantikine ELISA Kit (R&D Systems, Minneapolis, MN, USA) for FINRISK and PRIME Belfast and using the Mercodia ELISA (Mercodia AB, Uppsala, Sweden) for KORA F4.

Baseline concentrations of fasting glucose were only available for FINRISK and KORA-F4. In FINRISK, fasting glucose was measured from serum or EDTA plasma using a hexokinase method (Hexokinase/G-6-PDH, Architect c8000, Abbott). The required fasting duration was 4 hours, and the majority of FINRISK participants fasted at least 4 hours but less than 8 hours. In KORA F4, fasting glucose was measured from serum after an overnight fasting of at least 8 hours on an enzymatic colorimetric method on a Dimension Vista 1500 instrument (Siemens Healthcare Diagnostics Inc., Newark, NJ, USA) or the GLUC3 assay on a Cobas c702 instrument (Roche Diagnostics GmbH, Mannheim, Germany).

Estimated glomerular filtration rate (eGFR) in all participating cohorts was estimated using the CKD-EPI formula with creatinine (1).

**Text S2. Procedure for the univariate Mendelian randomisation analysis**

In our study, we tried to satisfy the three main assumptions for a valid genetic instrumental variable (IV) in our Mendelian randomisation analysis. The three assumptions are: (A1) the IV must be associated with the exposure (relevance), (A2) the IV should be independent of the outcome conditional on the exposure and confounders (exclusion restriction), and (A3) the IV should not be associated with confounders of the exposure-outcome association (exchangeability) (2, 3). The A1 assumption is the only assumption that can be directly tested (2).

The A1 assumption was satisfied by including single nucleotide polymorphisms (SNPs) that are associated with C-terminal-proendothelin-1 (CT-proET-1) or mid-regional-proadrenomedullin (MR-proADM) at a *P*-value < 5E-8 as the IVs. The data were extracted from a published genome-wide association (GWA) study of European ancestry from Verweij et al. (4). The study consists of 3,444 GWA study discovery samples and 3,230 replication samples from the Prevention of Renal and Vascular End-Stage Disease (PREVEND) study. We identified 6 SNPs for each peptide at a *P*-value < 5E-8.

Violations of the A2 and A3 assumption can occur in case of horizontal pleiotropy that is a scenario where a SNP is associated with other variables on different causal pathways to the outcome (2, 3). We tried to meet the A2 and A3 assumptions by including only SNPs that are not in linkage disequilibrium with each other and are specific for either CT-proET-1 or MR-proADM. For each peptide, we identified 3 out of 6 SNPs that are not in linkage disequilibrium with each other, using the r^2^ cut-off of 0.1 to obtain independent SNPs. Out of these 3 independent SNPs for each peptide, only 1 SNP that is specific for CT-proET-1 (rs5370; gene: *EDN-1*) and 1 SNP that is specific for MR-proADM (rs2957692; gene: *ADM*). Thus, we only included one SNP as the IV for each peptide in our Mendelian randomisation analysis. Furthermore, violations of the A3 assumption can also occur in case of population stratification when the study sample includes subgroups with different genetic ancestries and thus, different allele frequencies (2). We tried to overcome this issue by focusing our Mendelian randomisation analysis on homogeneous ancestry groups, in this case we focused our analysis on individuals with European ancestry.

The estimates of the genetic associations between IVs and the risk of type 2 diabetes were extracted from meta-analyses of GWA studies of European ancestry on type 2 diabetes by Mahajan et al. (5) comprising 48,286 type 2 diabetes cases and 250,671 controls and by Bonàs-Guarch et al. (6) comprising 12,931 type 2 diabetes cases and 57,196 controls, depending on the data availability. We prioritized the meta-analysis by Mahajan et al. due to the large sample size. Before performing the Mendelian randomisation analyses, we made sure that the IV association estimates with each vasoactive peptide and with type 2 diabetes correspond to the same effect alleles.

To compute the Mendelian randomisation estimates we used the Wald ratio (7) compared the results with the likelihood-based method (8). For an easier interpretation, we reported the estimates with 95% confidence intervals on the odds ratio (OR) scale. *P*-values < 0.05 were considered statistically significant. Due to a very few numbers of IVs, we were not able to perform other robust methods, including MR‐Egger regression model (9). Our Mendelian randomisation analyses were performed in the statistical software R version 4.0.3 (10) using “MendelianRandomization” R-package version 0.5.0 (11).

**References**

1. Levey AS, Stevens LA, Schmid CH, Zhang YL, Castro AF, 3rd, Feldman HI, et al. A new equation to estimate glomerular filtration rate. Ann Intern Med. 2009;150(9):604-12.

2. Haycock PC, Burgess S, Wade KH, Bowden J, Relton C, Davey Smith G. Best (but oft-forgotten) practices: the design, analysis, and interpretation of Mendelian randomization studies. Am J Clin Nutr. 2016;103(4):965-78.

3. Burgess S, Davey Smith G, Davies NM, Dudbridge F, Gill D, Glymour MM, et al. Guidelines for performing Mendelian randomization investigations. Wellcome Open Res. 2020;4:186.

4. Verweij N, Mahmud H, Mateo Leach I, de Boer RA, Brouwers FP, Yu H, et al. Genome-wide association study on plasma levels of midregional-proadrenomedullin and C-terminal-pro-endothelin-1. Hypertension. 2013;61(3):602-8.

5. Mahajan A, Wessel J, Willems SM, Zhao W, Robertson NR, Chu AY, et al. Refining the accuracy of validated target identification through coding variant fine-mapping in type 2 diabetes. Nat Genet. 2018;50(4):559-71.

6. Bonàs-Guarch S, Guindo-Martínez M, Miguel-Escalada I, Grarup N, Sebastian D, Rodriguez-Fos E, et al. Re-analysis of public genetic data reveals a rare X-chromosomal variant associated with type 2 diabetes. Nat Commun. 2018;9(1):321.

7. Burgess S, Small DS, Thompson SG. A review of instrumental variable estimators for Mendelian randomization. Stat Methods Med Res. 2017;26(5):2333-55.

8. Burgess S, Butterworth A, Thompson SG. Mendelian randomization analysis with multiple genetic variants using summarized data. Genet Epidemiol. 2013;37(7):658-65.

9. Bowden J, Davey Smith G, Burgess S. Mendelian randomization with invalid instruments: effect estimation and bias detection through Egger regression. Int J Epidemiol. 2015;44(2):512-25.

10. R Core Team. R: A Language and environment for statistical computing. Vienna, Austria: R Foundation for Statistical Computing; 2020.

11. Yavorska OO, Burgess S. MendelianRandomization: an R package for performing Mendelian randomization analyses using summarized data. Int J Epidemiol. 2017;46(6):1734-9.

The BiomarCaRE Consortium website: <http://www.biomarcare.eu/>

Sites and key personnel of contributing BiomarCaRE centers:

| **BiomarCaRE center** | **Investigators** |
| --- | --- |
| FINRISK | Veikko Salomaa (Principal Investigator), Anne Juolevi, Erkki Vartiainen, Pekka Jousilahti, Tiina Laatikainen, Kennet Harald. |
| Prospective Epidemiological Study of Myocardial Infarction (PRIME) Belfast | Frank Kee (Principal Investigator), Alun Evans (former Principal Investigator), John Yarnell, Angela Scott, Evelyn Gardner (up to 2008). |
| Kooperative Gesundheits-forschung in der Region Augsburg (KORA) | Barbara Thorand (Principal Investigator), Annette Peters (former Principal Investigator), Angela Döring (former Principal Investigator), Christa Meisinger, Margit Heier, Andrea Schneider. |
| BiomarCaRE Laboratory | Tanja Zeller (Head), Stefan Blankenberg, University Medical Center Hamburg-Eppendorf, Hamburg, Germany. |
| MORGAM Data Centre | Kari Kuulasmaa (Head), Ari Haukijärvi, Teemu Niiranen, Tarja Palosaari, Jaakko Reinikainen, Finnish Institute for Health and Welfare (THL), Helsinki, Finland |
| MORGAM/BiomarCaRE Steering Group | Stefan Blankenberg, Alun Evans, Licia Iacoviello, Frank Kee, Wolfgang Koenig, Kari Kuulasmaa, Teemu Niiranen, Markus Perola, Veikko Salomaa, Renate Schnabel, Hugh Tunstall-Pedoe, Giovanni Veronesi, Tanja Zeller. |
